# Breast tuberculosis: A case report of primary type mammary tuberculosis

**DOI:** 10.1002/ccr3.2486

**Published:** 2019-10-23

**Authors:** Giovanni Tazzioli, Antonella Macolino, Francesca Combi, Enza Palma, Simona Papi, Mauro Codeluppi, Cristina Mussini

**Affiliations:** ^1^ Breast Surgery Unit University Hospital of Modena Azienda Ospedaliero Universitaria Policlinico di Modena Modena Italy; ^2^ Clinic of Infectious Diseases University Hospital of Modena Azienda Ospedaliero Universitaria Policlinico di Modena Modena Italy

**Keywords:** breast abscesses, breast tuberculosis, granulomatous mastitis, QuantiFERON‐TB

## Abstract

Mammary tuberculosis is exceptional in developed countries. It can mimic an abscess or a granulomatous mastitis. In subjects coming from endemic areas, it is necessary to suspect a tuberculosis infection in case of recurrent mastitis refractory to antibiotics. Positivity of Quantiferon‐TB Gold assay can help to confirm the clinical suspicion.

## Graphical abstract

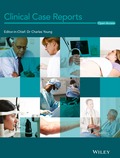

## 1 INTRODUCTION

Breast tuberculosis is a rare condition, especially as primary manifestation.^1^ The incidence is estimated to be 0.1% of breast diseases in developed countries, but it reaches 3%‐4% in countries where tuberculosis is endemic.^2^, ^3^, ^4^ Mammary tuberculosis mostly appears in multiparous, lactating women and in association with immunosuppressive disorders, including HIV.^5^, ^6^ The diagnosis can be difficult because the disease may mimic breast carcinoma, pyogenic abscess, and other granulomatous diseases.^7^, ^8^

## 2 CASE REPORT

A 31‐year‐old Chinese woman without chronic diseases and without familiar history of breast cancer was sent from Radiology to the Breast Surgery Department showing pain and purulent discharge from the right nipple. The cytological test on the material showed neutrophils and macrophages; ultrasound showed a centimetric hypoechoic subareolar lesion, as for a breast abscess. The patient was treated with empirical antibiotic therapy (Amoxicillin and Clavulanic Acid 1g every 12 hours for 6 days) with regression of symptoms. After three months the nipple discharge reappeared. Ultrasound showed only a ductal ectasia. The total leukocyte count and other blood tests were normal. The patient underwent again antibiotic treatment together with surgical right ductgalactophorectomy and toilette of the abscess. The histology of the specimen confirmed ductal ectasia, acute and chronic inflammation. After surgery a leak of dense material persisted. A culture was performed and an *Escherichia Coli* ESBL + grew up. The infectious diseases specialist prescribed a specific antibiotic therapy with IV Ertapenem 1 g/day for 7 days, which led to regression of symptoms. In the following two months, the patient was treated for several episodes of breast inflammation and purulent nipple leak. She underwent antibiotic therapy with Piperacilline + Tazobactam (4.5 g 3× per day for 7 days) and then ertapenem again with the same posology, and surgical toilette of the region was repeated twice. Nevertheless, the purulent secretion persisted, so we considered breast tuberculosis in differential diagnosis. QuantiFERON‐TB Gold assay was positive (presence of cell‐mediated immune response to antigens ESAT‐6 and CFP‐10 of *Mycobacterium tuberculosis*), but it was not possible to identify the *Mycobacterium tuberculosis* in the specimens of the wound. A chest X‐ray was negative, but because of the clinical nonresponse to antibiotic and of the results of QuantiFERON‐TB, a diagnosis of primary breast tuberculosis was made. The patient was treated with antitubercular drugs (rifampicin, isoniazid, pyrazinamide, and ethambutol) for three months, and she continued rifampicin and isoniazid for three additional months. In the follow‐up period of one year, there was no evidence of persistent disease.

## 3 DISCUSSION

BT is an extremely rare disease, first described in 1829.^9^ It can be primary (tuberculosis affects only the breast) and secondary (tuberculosis affects other organs).^10^ According to the latest classification, BT can present as nodular, disseminated, and tubercular abscess. The nodular type presents as a well‐defined mass that progressively involves the skin, sometimes causing ulceration. It is more frequent in the elderly. The disseminated type presents as multiple foci that can lead to confluence. The tubercular abscess is characterized by a cavity similar to other bacterial abscess, but that can later caseate.^5^ Our patient had a tubercular abscess.

In the diagnosis, mammography has low sensitivity. Ultrasound can show a hypoechoic mass in 60% of patients, focal or sectorial duct ectasia in 40%.^11^ The gold standard for the diagnosis is detection of *M tuberculosis* by Ziehl‐Neelsen staining or by culture. Fine needle aspiration cytology (FNAC) is generally performed to detect the bacilli by the stains or the presence of epithelioid cells and granulomas.^12^ In our report, a FNAC was performed but was not diagnostic. FNAC is typically inconclusive, and the acid‐fast bacilli are usually not seen even in culture.^13^ Histopathology of the lesion can be useful to identify chronic granulomatous inflammation with caseous necrosis.^14^ In our case, the clinical diagnosis was only supported by the positivity of QuantiFERON‐TB Gold assay.

It is important to consider BT in differential diagnosis with breast carcinoma, breast abscess that do not regress with classical nontubercular antibiotic therapy and other granulomatous mastitis,^15^ especially in patients coming from countries where the infection is endemic and in patients with immunodeficiency. A multidrug‐resistant infection should be excluded.

Since the disease could represent a diagnostic challenge, the research of *M Tuberculosis* either by histology or by culture is mandatory in patients at risk.

The treatment of breast tuberculosis is medical with antitubercular drugs.^16^ The surgical drainage and resection of the ducts alone result ineffective.

## CONFLICT OF INTEREST

None declared.

## AUTHOR CONTRIBUTIONS

Tazzioli Giovanni: involved in conception and design of the work. Macolino Antonella: involved in manuscript preparation and data acquisition. Combi Francesca: involved in manuscript preparation and data acquisition. Palma Enza: involved in manuscript review and clinical management. Papi Simona: involved in manuscript review and clinical management. Codeluppi Mauro: involved in clinical management. Mussini Cristina: involved in conception of the work and manuscript review.
